# Steal syndrome from a superficial circumflex iliac perforator artery flap in a patient with a hypoplastic posterior tibial artery and severe diabetic peripheral artery disease

**DOI:** 10.1093/jscr/rjab067

**Published:** 2021-03-22

**Authors:** Grant A Murphy, Rajinder P Singh-Moon, Vincent L Rowe, Ketan M Patel, Amaan Mazhar, David J Cuccia, David G Armstrong

**Affiliations:** Southwestern Academic Limb Salvage Alliance (SALSA), Department of Surgery, Keck School of Medicine, University of Southern California, Los Angeles, CA, USA; Department of Research and Development, Modulim, Irvine, CA, USA; Division of Vascular Surgery, Department of Surgery, Keck School of Medicine, University of Southern California, Los Angeles, CA, USA; Division of Plastic and Reconstructive Surgery, Department of Surgery, Keck School of Medicine, University of Southern California, Los Angeles, CA, USA; Department of Research and Development, Modulim, Irvine, CA, USA; Department of Research and Development, Modulim, Irvine, CA, USA; Southwestern Academic Limb Salvage Alliance (SALSA), Department of Surgery, Keck School of Medicine, University of Southern California, Los Angeles, CA, USA

## Abstract

The use of free flaps in lower extremity reconstructive surgery has seen growing adoption for treating tissue loss in patients with diabetes mellitus and peripheral artery disease as a means for limb preservation. The superficial circumflex iliac perforator artery (SCIP) flap is one of the most commonly utilized flaps in foot reconstruction and has demonstrated benefits over amputation. Patients with impaired vascular and neurologic function are predisposed to complications following lower extremity reconstructive surgery, particularly ischemia in the angiosomes of the arteries used for flap anastomosis. We present the case of a patient who underwent successful SCIP flap reconstruction of the calcaneus but developed gangrene in the forefoot region supplied by a hypoplastic posterior tibial artery in subsequent months. The changes in tissue oxygenation and hemoglobin distribution of the foot are shown using spatial frequency domain imaging throughout the flap healing process and eventual tissue necrosis.

## INTRODUCTION

Treating lower extremity tissue loss with free flaps has become an increasingly utilized approach for limb preservation in patients with diabetic foot ulcers (DFU; [1]). Free flap reconstructive surgery allows for correction of foot defects and has shown improved 5-year survival when compared to above-ankle amputation in patients with diabetes mellitus (DM; [2, 3]). However, patients with PAD and anomalous vascular anatomy may be susceptible to limb complications arising from the hemodynamic changes following free flap surgeries.

We present the case of a patient with left calcaneal tissue loss who underwent superficial circumflex iliac perforator artery (SCIP) flap reconstruction and eventual forefoot amputation secondary to gangrenous complications. Alongside conventional care, observational plantar measurements of microvascular oxygenation (StO_2_) and hemoglobin (Hb) distribution were captured throughout the treatment course using a commercial spatial frequency domain imaging (SFDI) system (Research RS, Modulim, Irvine, CA, USA) [4]. SFDI data were retrospectively analyzed to ascertain early indications which may prognosticate vascular steal.

## CASE REPORT

A very active 58-year-old man with type 2 DM and severe PAD presented with a 6.0 × 5.0 cm full-thickness neuroischemic DFU on the left calcaneus. The patient was initially offered an above-knee amputation, as there was concern he would be unable to heal a below-knee amputation due to severe PAD. The patient subsequently sought treatment at a comprehensive limb preservation clinic and received multidisciplinary wound care for concomitant tissue loss and PAD [5].

**Figure 1:**
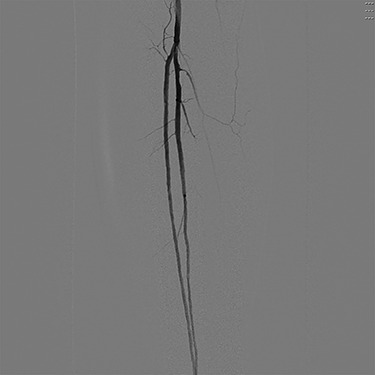
Digital subtraction angiography of the left popliteal artery and its tibial branches, demonstrating a hypoplastic posterior tibial artery.

**Figure 2:**
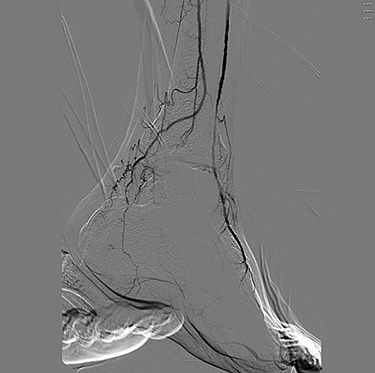
Digital subtraction angiography of the left pedal vessels.

Angiography (Figs 1 and 2) demonstrated bilateral hypoplasia of the posterior tibial arteries (PTA), with the anterior tibial artery supplying most of the blood to the forefoot. The peroneal arteries were also noted to provide large collateral branches posteriorly and were the primary arterial supply to the heel. Additionally, the medial plantar artery (MPA) arising from the PTA was nearly absent and the plantar arch was incomplete bilaterally.

**Figure 3:**
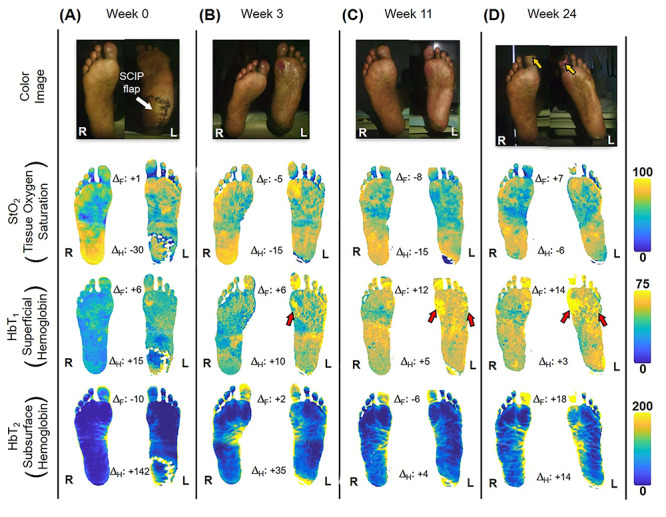
Quantitative longitudinal monitoring of plantar microvascular changes following SCIP flap reconstruction. SFDI-derived maps for StO_2_, HbT_1_ and HbT_2_ are shown at (**A**) Week 0, (**B**) Week 3, (**C**) Week 11 and (**D**) Week 24 following surgery. **Δ**_**F**_ and **Δ**_**H**_ denote bilateral differences (left–right) between forefoot (F) and heel (H) mean values. The yellow arrows indicate worsening gangrene at the halluces. Red arrows indicate regions with a blotchy distribution pattern.

**Figure 4:**
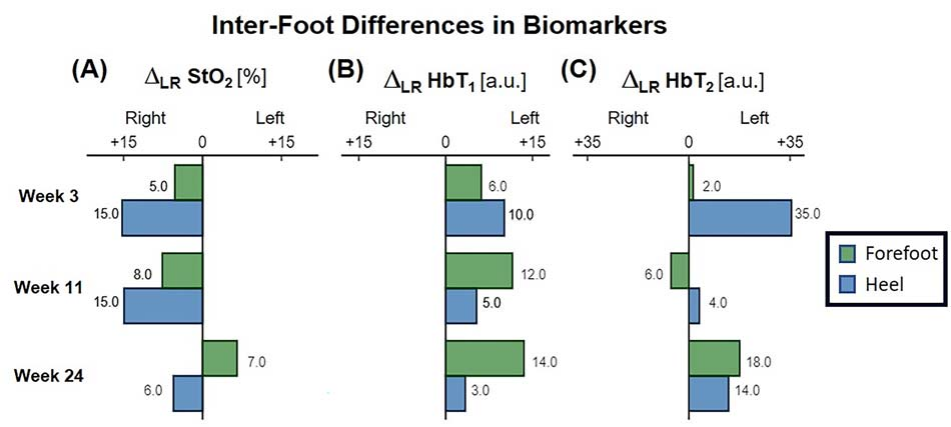
Bar plot depicting trends in inter-foot asymmetry for SFDI-derived biomarkers, separated by region. **Δ**_**LR**_ denotes the bilateral difference in mean pixel value computed for forefeet and heel regions.

**Figure 5:**
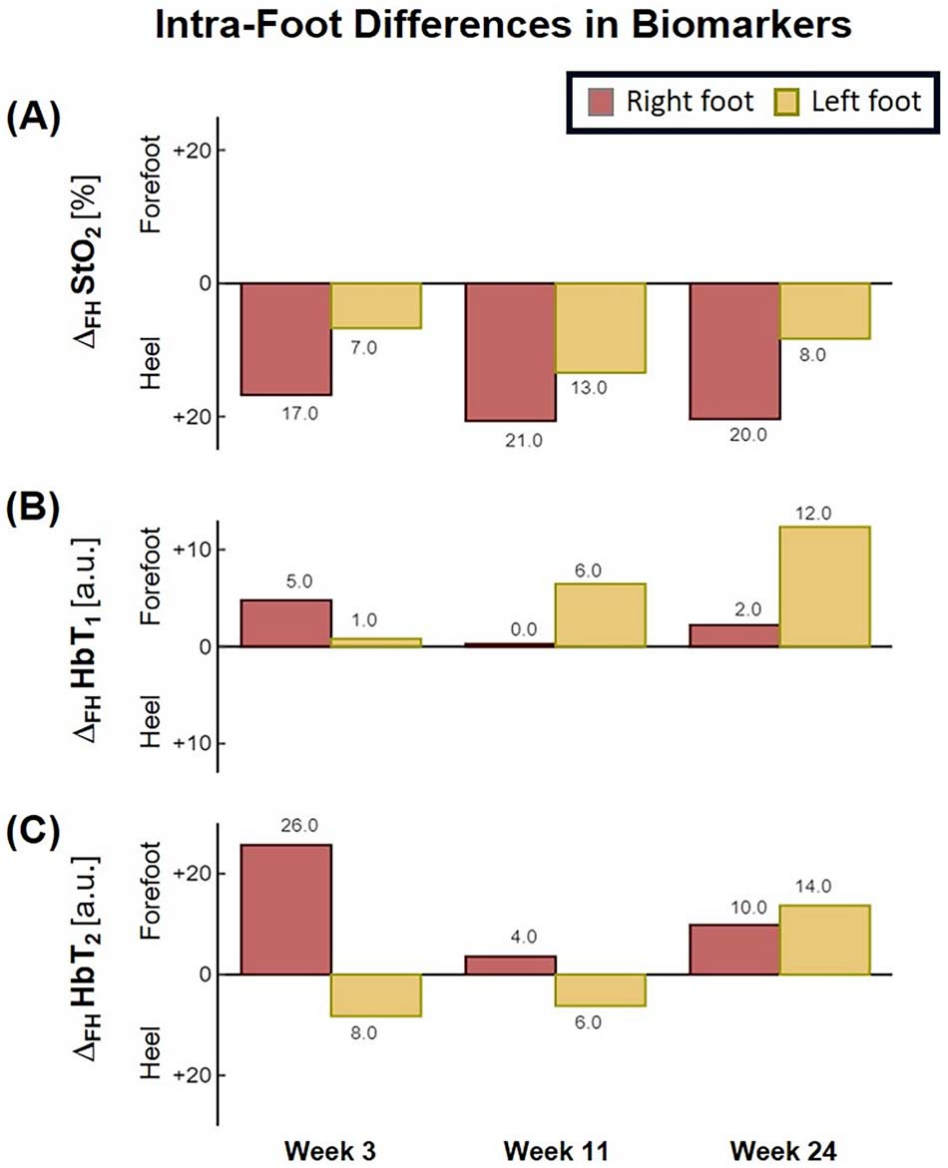
Bar plot depicting trends in intra-foot asymmetry for SFDI-derived biomarkers, separated by foot. **Δ**_**FH**_ denotes the difference between forefoot and heel averaged pixel values.

The patient first underwent a debridement and left partial calcanectomy. The calcanectomy grew out Candida species with a clean margin both histologically and microbiologically. Three weeks later the patient then underwent a SCIP flap reconstruction of left heel, which was anastomosed to the PTA in an end-side fashion. Intraoperative flap assessment demonstrated adequate arterial perfusion and venous drainage. SFDI-derived StO_2_, along with superficial papillary Hb (HbT_1_) and deep reticular Hb (HbT_2_) measurements throughout the treatment course are shown in Fig. 3. Postoperative mean pixel values for flap and surrounding plantar regions of the left foot both demonstrated decreased StO_2_ (−35%) compared to the contralateral foot (Fig. 3A); comparatively greater HbT_1_ (+54%) and HbT_2_ (+887%) density at the flap margins was also noted (Fig. 3A). As time progressed, a steady trend of reduced inter-foot (left/right) heel asymmetry was observed over all biomarkers (Figs 3B–D and4), continuing to persist after full incorporation of the flap was noted at 9 weeks following reconstruction (Fig. 4A and B). Conversely, inter-foot forefoot differences in Hb distribution widened, starting off negligible and gradually evolving between Weeks 3 and 24 (HbT_1_ from +14 to +25% and HbT_2_ from +2 to +22%) (Figs 3 and 4). Though intra-foot similarities such as lower forefoot StO_2_ (Fig. 5A) and elevated hallux HbT_1_ and HbT_2_ (Fig. 3) were present bilaterally, the difference between mean forefoot and heel HbT_1_ grew more pronounced in the left foot (+2 to +22% relative to heel) compared to the right (+12 to +4% relative to heel) (Fig. 5B). This tracked with an increasing prevalence of visible hotspots in left foot HbT_1_ maps, dispersed throughout the digital and metatarsal regions (Fig. 3B–D, third row).

The patient would go on to develop bilateral neuroischemic ulcers of the distal halluces 14 weeks after reconstruction. While the right hallux wound stabilized, the left forefoot became increasingly gangrenous, which necessitated open transmetatarsal amputation (TMA) at 44-week post-reconstruction.

## DISCUSSION

Tissue necrosis within the angiosome of the flap arterial supply following foot reconstruction has been previously described in the literature [6, 7]. In addition to the patient’s PAD, variant anatomy likely contributed to the forefoot gangrene, as the incomplete plantar arterial arch prevented collateral circulation between the hypoplastic PTA and anterior tibial artery. SCIP flaps contain highly vascular tissue [8] and without collateral circulation from the plantar arch, the left forefoot became critically ischemic in the months following the addition of the flap. Of note, an absent or hypoplastic PTA is one of the more common lower extremity vascular variants, comprising over 5% of patients in a review of 1242 angiograms [9].

With bilateral neuroischemic hallux ulceration and toe pressures of 0 mmHg, both medial forefeet clearly had poor arterial supply. While the right forefoot ultimately recovered with minimal tissue loss, the addition of the flap to the PTA likely contributed to the more extensive tissue loss seen on the left side, as the stenotic vessels were unable to compensate for decreased blood flow when the flap was added in parallel to the poorly perfused tissue. In retrospect, consequential microcirculatory changes appear to be detectable by SFDI prior to clinical presentation of progressive ischemia and necrosis. Namely, the divergent biomarker patterns of reduced heel asymmetry and increased forefoot asymmetry with respect to the contralateral foot may be indications of SCIP flap incorporation and survival alongside concurrent left forefoot ischemia, respectively. Moreover, the blotchy HbT_1_ distribution pattern may be early signs of mottling; a phenomenon commonly reported to precede accelerated ischemic changes (Fig. 3, red arrows) [6, 10]. Relative to heel values, the reduced forefoot StO_2_ in concert with increased HbT_1_ and HbT_2_ may suggest stasis of deoxygenated blood within the dermis, likely a result of low pressure and flow within the vessels supplying the distal foot (Fig. 5; [4]). This case demonstrates how microvascular assessments of oxygenation and perfusion, mediated by SFDI, may provide time-critical insights into precursory ischemic signatures and can serve as a valuable adjunct alongside conventional vascular testing.

Patients with tissue loss from diabetic neuropathy and PAD who may benefit from flap reconstructions must undergo thorough vascular assessment as part of preoperative planning to avoid inadvertent compromise of lower extremity angiosomes. The complications sustained by this patient should be taken into consideration for others with similar anatomy and comorbidities, as the factors that necessitate lower extremity flap reconstruction are the same that complicate its use.

## CONFLICT OF INTEREST STATEMENT

Rajinder P. Singh-Moon, Amaan Mazhar and David J. Cuccia are full time employees of Modulim and are commercializing SFDI technology.

## FUNDING

This study is partially supported by National Institutes of Health, National Institute of Diabetes and Digestive and Kidney Diseases Award Number 1R01124789-01A1.
